# Supplementary material for: Flexible, scalable, high channel count stereo-electrode for recording in the human brain
Source: Nat Commun. 2024 Jan 17;15:218. doi: 10.1038/s41467-023-43727-9 (PMC10794240; doi:10.1038/s41467-023-43727-9)
Supplement: Supplementary file 3 — Description of Additional Supplementary Files [file 41467_2023_43727_MOESM3_ESM.pdf]

Description of Additional Supplementary Files

File Name: Supplementary Video 1
Description: **Movement of the implanted short thin film µSEEG relative to the brain.** The electrode is shown before and after insertion, where the short depth was inserted into brain tissue and activity was recorded for a short period of time. The gauze and other stabilizing features are largely for the ribbon cables which form the connections, but the thin film components (which are largely transparent and flexible) move with the brain tissue movements in the craniotomy.
