# Supplementary material for: Flexible, scalable, high channel count stereo-electrode for recording in the human brain
Source: Nat Commun. 2024 Jan 17;15:218. doi: 10.1038/s41467-023-43727-9 (PMC10794240; doi:10.1038/s41467-023-43727-9)
Supplement: Supplementary file 5 — Reporting Summary [file 41467_2023_43727_MOESM5_ESM.pdf]

nature portfolio

Corresponding author(s): Shadi Dayeh

Last updated by author(s): Oct 29, 2023

Reporting Summary

Nature Portfolio wishes to improve the reproducibility of the work that we publish. This form provides structure for consistency and transparency in reporting. For further information on Nature Portfolio policies, see our Editorial Policies and the Editorial Policy Checklist.

Statistics

For all statistical analyses, confirm that the following items are present in the figure legend, table legend, main text, or Methods section.

n/a	Confirmed	
☐	☒	The exact sample size (*n*) for each experimental group/condition, given as a discrete number and unit of measurement
☐	☒	A statement on whether measurements were taken from distinct samples or whether the same sample was measured repeatedly
☐	☒	The statistical test(s) used AND whether they are one- or two-sided<br>*Only common tests should be described solely by name; describe more complex techniques in the Methods section.*
☐	☒	A description of all covariates tested
☐	☒	A description of any assumptions or corrections, such as tests of normality and adjustment for multiple comparisons
☐	☒	A full description of the statistical parameters including central tendency (e.g. means) or other basic estimates (e.g. regression coefficient) AND variation (e.g. standard deviation) or associated estimates of uncertainty (e.g. confidence intervals)
☐	☒	For null hypothesis testing, the test statistic (e.g. *F*, *t*, *r*) with confidence intervals, effect sizes, degrees of freedom and *P* value noted<br>*Give P values as exact values whenever suitable.*
☒	☐	For Bayesian analysis, information on the choice of priors and Markov chain Monte Carlo settings
☒	☐	For hierarchical and complex designs, identification of the appropriate level for tests and full reporting of outcomes
☐	☒	Estimates of effect sizes (e.g. Cohen's *d*, Pearson's *r*), indicating how they were calculated

Our web collection on statistics for biologists contains articles on many of the points above.

Software and code

Policy information about availability of computer code

Data collection	OpenEphys (http://www.open-ephys.org/) and Intan software (https://intantech.com/RHX_software.html)
Data analysis	Most of data were extracted and processed using MATLAB (Mathworks, Natick, MA). Custom MATLAB code (version R2021a) are available in GitHub (https://github.com/Center-For-Neurotechnology/MicrosEEG_Data_Analysis, Zenodo DOI: 10.5281/zenodo.10042080). Spike sorting was performed using kilosort 2.5 (https://github.com/MouseLand/Kilosort) with further determination of single units versus multi-unit activity performed in post processing using Phy (https://github.com/cortex-lab/phy) and then manually curated using in-house MATLAB code to visually inspect the template as well as the waveforms assigned to each cluster. We detected bursts and calculated the burst suppression ratio (BSR) using an automated method (https://github.com/drasros/bs_detector_icueeg). To visualize the locations in 3D in the non-human primate and rodent brains, we used the Scalable Brain Atlas with the Calabrese atlas (https://scalablebrainatlas.incf.org/; exported into Blender (https://www.blender.org/). For all clusters, we measured the spike duration, peak-trough ratio, and amplitude measures (Fig. 4; code adapted from https://github.com/jiaxx/waveform_classification).

For manuscripts utilizing custom algorithms or software that are central to the research but not yet described in published literature, software must be made available to editors and reviewers. We strongly encourage code deposition in a community repository (e.g. GitHub). See the Nature Portfolio guidelines for submitting code & software for further information.

Data

Policy information about availability of data

All manuscripts must include a data availability statement. This statement should provide the following information, where applicable:

- Accession codes, unique identifiers, or web links for publicly available datasets
- A description of any restrictions on data availability
- For clinical datasets or third party data, please ensure that the statement adheres to our policy

All data obtained in this study are either presented in the paper and the Supplementary Materials or deposited in open database. Animal brain recording data could be accessed at OpenNeuro ((https://openneuro.org/ at OpenNeuro Accession Number ds004819), and the human brain recording data could be found in Data Archive BRAIN Initiative (DABI) (https://dabi.loni.usc.edu/ at https://doi.org/10.18120/dn61-9y73) using the iEEG BIDS format.

Human research participants

Policy information about studies involving human research participants and Sex and Gender in Research.

Reporting on sex and gender	N=2 individuals, sex distribution includes 1 male and 1 female<br>Sex and gender were not considered in sampling the data or in collecting information. As each case in the operating room was also independently gathered to test the feasibility of using these devices for recording human brain activity, we did not compare activity between cases.
Population characteristics	The patients were approached following already being scheduled to remove a portion of the left anterior temporal lobe for the treatment of epilepsy. Recordings in the operating room were acquired with 2 participants (age 28 and 46; 1 female; 1 male; Supplemental Table 1) who were already scheduled for a craniotomy for concurrent clinical intraoperative neurophysiological monitoring or testing for mapping motor, language, and sensory regions and removal of tissue as a result of epilepsy.
Recruitment	Intraoperative recordings were performed in two participants who underwent a neurosurgical procedure at Massachusetts General Hospital (MGH). Both participants were involved voluntarily, provided informed consent, and were informed that participation in the experiment would not alter their clinical treatment in any way and that they could withdraw at any time without altering their clinical care. The patients were approached following already being scheduled to remove a portion of the left anterior temporal lobe for the treatment of epilepsy. The possibility of conducting research recordings were only discussed with each patient after the decision to proceed with the surgery had been made. Neither patient was medically unstable or required emergency or urgent surgery. All decisions to proceed were made following consultation with the treating neurosurgeon and clinical team. Further, we did not enroll patients with clearly impaired decision-making abilities (as determined by the primary clinical team or physician caring for the patient). The time allowed for dedicated research recording was limited per subject to minimize risk as well. Finally, we only asked the short SEEG electrodes could be inserted into tissue the clinical team identified would be resected following recording. There were no selection biases other than the clinical constraints of being cases where depth electrode could be inserted into tissue known to be resected later in the surgery.
Ethics oversight	This study was approved by the Partners Institutional Review Board (now the Mass General Brigham Institutional Review Board), which covers MGH.

Note that full information on the approval of the study protocol must also be provided in the manuscript.

Field-specific reporting

Please select the one below that is the best fit for your research. If you are not sure, read the appropriate sections before making your selection.

☒ Life sciences ☐ Behavioural & social sciences ☐ Ecological, evolutionary & environmental sciences

For a reference copy of the document with all sections, see nature.com/documents/nr-reporting-summary-flat.pdf

Life sciences study design

All studies must disclose on these points even when the disclosure is negative.

Sample size	Sample size was determined on the per-participant data set which was a total of N=2 participants. The sample sizes were determined by the availability of the cases and based on patient and clinical consent. There was no sample size calculation performed as conditions for electrode testing in the human operating room required a number of conditions were met (see above).
Data exclusions	Data exclusions were based on the quality of the neural signal and if the probe could be used in the case.
Replication	This is a technical report detailing the capability of microSEEG devices to capture neural activity, we report only the recordings from the cortex of temporal and frontal lobes in patients undergoing brain tissue resection to treat epilepsy (N=1, under general anesthesia, lateral

	temporal lobe) or during the resection of tumor tissue (N=1, one under monitored anesthesia care and the patient was awake, lateral prefrontal cortex) using microSEEG devices. We do not have further replications in our study design.
Randomization	Randomization was not relevant to the study as this was a study performed in the human operating room and depended on patient consent and recording during different conditions taking into account clinical considerations per participant. In addition, this was not a clinical trial examining treatment effects so much as a technical report to demonstrate the feasibility to record in the human brain using a Neuropixels probe. Therefore, randomization is not relevant for these experiments.
Blinding	Human data were de-identified and assigned a unique numerical code. Subsequent preprocessing were performed blinded to the conditions of the data acquisition though in once case, the data involved examining auditory cues and the other involved examining anesthesia-induced brain activity changes which could not be blinded to the case conditions. Blinding was therefore not possible.

Reporting for specific materials, systems and methods

We require information from authors about some types of materials, experimental systems and methods used in many studies. Here, indicate whether each material, system or method listed is relevant to your study. If you are not sure if a list item applies to your research, read the appropriate section before selecting a response.

Materials & experimental systems

n/a	Involved in the study	
☒	☐	Antibodies
☒	☐	Eukaryotic cell lines
☒	☐	Palaeontology and archaeology
☐	☒	Animals and other organisms
☒	☐	Clinical data
☒	☐	Dual use research of concern

Methods

n/a	Involved in the study	
☒	☐	ChIP-seq
☒	☐	Flow cytometry
☒	☐	MRI-based neuroimaging

Animals and other research organisms

Policy information about studies involving animals; ARRIVE guidelines recommended for reporting animal research, and Sex and Gender in Research

Laboratory animals	Rats were 3 months old, male, Sprague-Dawley. Pigs were 7 months old, female, Yorkshire. Non-human Primates were two adult male rhesus macaques (ages 11 and 14, Macaca mulatta)
Wild animals	The study didn't involve wild animals
Reporting on sex	All rats were male.<br>Pigs were female.<br>Both non-human primates (NHPs) were male.
Field-collected samples	None
Ethics oversight	All rodent and pig experiments were approved by the UCSD Institutional Animal Care and Use Committee (IACUC, protocol # S19030). For NHPs, all efforts were made to minimize discomfort, and the Institutional Animal Care and Use Committee at the Massachusetts General Hospital monitored care and approved all procedures.

Note that full information on the approval of the study protocol must also be provided in the manuscript.
